# Sema3A promotes the resolution of cardiac inflammation after myocardial infarction

**DOI:** 10.1007/s00395-017-0630-5

**Published:** 2017-05-24

**Authors:** Marieke Rienks, Paolo Carai, Nicole Bitsch, Mark Schellings, Maarten Vanhaverbeke, Johan Verjans, Ilona Cuijpers, Stephane Heymans, Anna Papageorgiou

**Affiliations:** 1 Cardiovascular Research Institute Maastricht, Universiteitssingel 50, 6229 ER Maastricht, The Netherlands; 2 Department of Cardiovascular Sciences, KU Leuven, Leuven, Belgium; 3 ICIN-Netherlands Heart Institute, Utrecht, The Netherlands; 4 University Medical Center Utrecht, Heidelberglaan 100, 3584 CX Utrecht, The Netherlands; 5 South Australian Health and Medical Research Institute, University of Adelaide, North Terrace, Adelaide, SA 5000 Australia

**Keywords:** Myocardial infarction, Inflammation, Heart failure, Semaphorin3A

## Abstract

Optimal healing after myocardial infarction requires not only the induction of inflammation, but also its timely resolution. In patients, 30 days post myocardial infarction, circulating monocytes have increased expression of Semaphorin3A (Sema3A) as compared to directly after admission. This increased expression coincides with increased expression of Cx3CR1—a marker of non-classical monocytes that are important for immune resolution hence proper wound healing. In mice, the expression of Sema3A also increases in response to myocardial ischemia being expressed by infiltrating leukocytes. Comparing Sema3A heterozygote (HZ) and wild type (WT) mice post myocardial infarction, revealed increased presence of leukocytes in the cardiac tissues of HZ mice as compared to WT, with no differences in capillary density, collagen deposition, cardiomyocyte surface area, chemokine—or adhesion molecules expression. Whilst infarct sizes were similar 14 days after myocardial infarction in both genotypes, Sema3A HZ mice had thinner infarcts and reduced cardiac function as compared to their WT littermates. In vitro experiments were conducted to study the role of Sema3A in inflammation and resolution of inflammation as a potential explanation for the differences in leukocyte recruitment and cardiac function observed in our in vivo experiments. Here, recombinant Sema3A protein was able to affect the pro-inflammatory state of cultured bone marrow derived macrophages. First, the pro-inflammatory state was altered by the induced apoptosis of classical macrophages in the presence of Sema3A. Second, Sema3A promoted the polarization of classical macrophages to resolution-phase macrophages and enhanced their efferocytotic ability, findings that were reflected in the infarcted cardiac tissue of the Sema3A HZ mice. Finally, we demonstrated that besides promoting resolution of inflammation, Sema3A was also able to retard the migration of monocytes to the myocardium. Collectively our data demonstrate that Sema3A reduces cardiac inflammation and improves cardiac function after myocardial infarction by promoting the resolution of inflammation.

**Electronic supplementary material:**

The online version of this article (doi:10.1007/s00395-017-0630-5) contains supplementary material, which is available to authorized users.

## Introduction

The improved clinical management of acute myocardial infarction (AMI) has resulted in a substantial reduction in mortality, yet this has led to the increased prevalence of heart failure and represents a huge socio-economic burden [23]. Cardiac inflammation in response to ischemia is key for repairing the damaged myocardium yet sustained inflammation results in adverse remodeling and consequently poor cardiac function and prognosis [17, 35]. Thus, post-infarct inflammation promotes infarct healing granted in its containment via the timely clearance of recruited pro-inflammatory leukocytes [16, 32]. The signals that recruit pro-inflammatory monocytes (LyC^hi^) has been well characterized [30], but little is known about the factors that switch off inflammation [2]. Recent studies have introduced the possibility that the transition to repair is not solely driven by the recruitment of reparative monocytes from the circulation, but suggest that local factors may be a key in promoting resolution from within the heart [18]. Understanding the molecular mechanisms that underline the transition to repair is important in designing new effective therapeutic strategies.

Semaphorin3A (Sema3A) is a secreted glycoprotein first identified as a chemo-repellent during axonal guidance [26], and crucial for embryonic development [3, 10]. In addition to its involvement in cell motility [12] and neuronal-vascular growth [15, 37], Sema3A has also been ascribed with tumor-reducing capacities [8] and anti-inflammatory properties during dermatitis [31], autoimmune arthritis [6] and kidney injury [33]. Sema3A is also a potent suppressor of T-lymphocyte activity [7, 24, 25, 29]. In the failing heart, Sema3A has been shown to be expressed by cardiomyocytes and to contribute to the sympathetic denervation in chronic heart failure [9], yet its function during cardiac inflammation and wound healing is unknown. In this study, we identify Sema3A as a key factor expressed by infiltrating leukocytes that promote the resolution of inflammation thereby facilitating cardiac wound healing and improving cardiac function.

## Materials and methods

### Human monocyte samples

We prospectively collected blood samples from 11 patients with acute coronary syndrome (ACS) on admission, at 3 days and at 30 days. Patients with cardiogenic shock, end-stage renal disease (eGFR <15 ml/min), inflammatory disease or history of malignancy were excluded. Patient characteristics can be found in Supplementary Table 1. The study protocol was approved by the institution’s ethical committee (Ethical Committee ML8525, Belgian trial no. B322201214942, S54129, September 2012) and all patients provided informed consent. During hospitalization, high sensitivity troponin T and hsCRP were measured. Monocytes were isolated by Ficoll density gradient centrifugation of citrated whole blood, followed by CD14^+^ magnetic beads selection of the mononuclear fraction (Miltenyi). Total RNA was extracted using the Qiagen miRNeasy mini kit, and the RNA yield and integrity was determined with NanoDrop spectrophotometer.

### Animals

For this study Sema3A heterozygote knock out (HZ) mice and wild type (WT) mice were used, and were a gift from Dr. J. Verhaagen, Netherlands Institute for Neuroscience, Amsterdam. As Sema3A is a vital protein for proper embryonic development of the nervous system [3], we noticed that the born homozygote knockout mice presented with severe retardation and died within a few days after birth, as was also described by Taniguchi et al. that generated these Sema3A knock out mice [36]. Therefore, we bred the colony by crossing Sema3A wild type with Sema3A heterozygote mice, to prevent the birth of these severely impaired Sema3A knock out mice. All animal experiments were approved by the Animal Care Committees of Maastricht University (DEC 2007-051/2013-055) and Leuven University (ECD 232/2013). Mice were age-matched at the start of the experimental protocol and experiments were performed using both male and female mice at random.

### Myocardial infarction model

Myocardial infarction was induced by ligation of the left anterior descending artery after subcutaneously anaesthetizing the animals with a mix of ketamine xylazine, respectively, 100 and 5 mg/kg subcutaneously in a volume of 0.1 ml 0.9% NaCl/10 g bodyweight. After anesthetizing, the animals were intubated with a 20-gauge intra-tracheal cannula. Rectal temperature was constantly measured during the procedures. After shaving the animals, an incision was made microscopically. As a clear view of the left coronary artery is reached, the left anterior descending artery was ligated. After successful ligation of the left anterior descending artery, the wound was closed with soluble stitches. Sham operations included all procedures except ligation of the left anterior descending artery. Echocardiography was performed prior to killing at 2 weeks post myocardial infarction. Heart, lung, kidney, and liver weights were measured. The left ventricle was separated from the heart and divided for histological and molecular analyses. Heart tissue for molecular analyses was immediately placed in liquid nitrogen, and stored at −80 °C.

### Echocardiography

To gain echocardiographic images, the animals were anesthetized by ventilation with isoflurane 3–4%. The anesthesia was maintained by artificially ventilating with a mixture of O_2_ and N_2_O [1:2 (vol/vol)] to which isoflurane 1.5–2.5% was added. Standard views were obtained in 2-D as well as M-mode by transthoracic echocardiography using a 12 MHz probe (Hewlett Packard, Amsterdam, The Netherlands) on a Visual Sonics echocardiograph.

### FACS analysis

Peripheral blood samples were analyzed using a FACS CantoII flow cytometer (Becton Dickenson (BD), San Diego, CA, USA). Cells were first incubated with anti-CD16/32 (eBioscience, San Diego, CA, USA) to block Fc receptor binding. CD45^+^ leukocytes subpopulations were defined as follows: T lymphocytes (CD45^+^, CD3^+^, B220^−^), B lymphocytes (CD45^+^, CD3^−^, B220^+^), Granulocytes (CD45^+^, CD3^−^, B220^−^, CD11b^+^, Ly6G^+^), and Monocytes (CD45^+^, CD3^−^, B220^−^, CD11b^+^, Ly6G^−^, CD11b^+^, Ly6C-or^+^). Absolute counts were determined with BD Truecount™ tubes according to the manufacturer’s instructions.

### Staining and immunohistochemistry

Hearts were perfused from the apex with phosphate buffered saline (PBS), fixed overnight and processed the following day, before being embedded in paraffin. The embedded left ventricle was cut longitudinally in 4 μm sections to optimally demonstrate the infract area. Sections were stained with hematoxylin/eosin for detection of infarct size and thickness. Collagen was stained on cardiac sections using Sirius Red, and the amount of collagen was quantified. Immunohistochemistry on paraffin sections was performed according to protocol using antibodies against CD45 (leukocytes), CD68 or Mac-3 (macrophages), CD31 (endothelial cells), and Sema3A and quantified in the infarct area. Images were acquired using Leica Qwin image processing software (Leica, Germany).

### Western blot

Tissues were lysed in RIPA SDS (50 mM Tris–HCl, 150 mM NaCl, 0.1% SDS, 0.5% sodium deoxycholate, 1% NP40, Proteinase Inhibitor Cocktail, Roche, 11697498001 and 0.5 mM orthovandate) after which the protein concentration was determined using a Micro BCA Protein assay kit (Thermo Scientific, lot# MJ162220). Samples were diluted to a final concentration of 2 μg/μl after which two times sample buffer (25 ml 0.5 M Tris–HCl, 20 ml 100% glycerol, 20 ml, 20% SDS, 35 ml Aqua Dest with 1:10 β-mercaptoethanol) was added to protein samples 1:1. Cells were directly lysed in sample buffer. For western blot analyses 20 μg protein was loaded on a 10% gel (4 ml Aqua Dest, 3.3 ml 30% AcrylAmide, 2.5 ml 1.5 M Tris–HCl pH 8.8, 0.1 ml 10% SDS, 0.004 ml TEMED). SDS PAGE was performed at 150 V for approximately 90 min after which the gel was transferred to a PVDF membrane by blotting at 200 mA for 2 h. Membranes were blocked with 3.5% Protifar for 1 h. Overnight primary antibody incubation was performed in 5% BSA with antibodies against Sema3A (Abcam, ab23393), Cleaved Caspase 3 (Cell Signaling Technology (CST) #9664S), BCL2 (Cell Signaling Technology (CST) #2870S), GAPDH (Millipore, MAB374).

### RT-PCR

Real-time reverse transcriptase-polymerase chain reaction (RT-PCR or QPCR) analysis was performed (Bio-Rad, Maastricht, The Netherlands) to describe transcript levels of Sema3A (forward primer CGGTGGCTCAATGATCCTAGA, reverse primer TTTGTCATCTTCAGGGTTGTCACT at 63.9 °C), CVB3 (forward primer ACGAATCCCAGTGTGTTTTGG, reverse primer TGCTCAAAAACGGTATGGACAT at 63.9 °C), GAPDH (forward primer GGTGGACCTCATGGCCTACA, reverse primer CTCTCTTGCTCAGTGTCCTTGCT at 63.9 °C), VCAM1 (forward primer CCGGCATATACGAGTGTGAA, reverse primer GATGCGCAGTAGAGTGCAAG at 63.9 °C), ICAM1 (forward primer TGGAGACGCAGAGGACCTTA, reverse primer CGCTCAGAAGAACCACCTTC at 63.9 °C), PECAM1 (forward primer CCCCCAGAACATGGATGTAG, reverse primer GTCTCTGTGGCTCTCGTTCC at 63.9 °C), MCP1 (forward primer ACCAGCAGCAGGTGTCCC, reverse primer GCACAGACCTCTCTCTTGAGCTT at 63.9 °C), CCL7 (forward primer ACCAGTAGTCGGTGTCCCTG, reverse primer AGGCTTTGGAGTTGGGGTTTTCA at 63.9 °C), IL6 (forward primer CAAAGCCAGAGTCCTTCAGAG, reverse primer GCCACTCCTTCTGTGACTCC at 63.9 °C). The primers sequences of these genes were determined by NCBI software analysis of Primer BLAST. Details of sequences and thermal cycle conditions are according to protocol. Data were acquired and analyzed using IQ5 software (Bio-Rad, Maastricht, The Netherlands).

### Monocyte isolation from murine spleens

Spleens were collected from Sema3A WT and HZ mice and cut into small pieces. With the back of a syringe the spleen was pushed through a 70 µm Cell Strainer (BD Falcon), while rinsing with ice cold PBS. After collecting the cells in a 50 ml tube they were spun down. A single cell suspension was obtained by dissolving the cells in 1 ml ice cold PBS which was followed by manual counting of cell with the use of a Burker Turk Counting chamber. Cells were spun down again and dissolved in the appropriate volume of PBS for MACS miltenyi monocyte isolation. Monocytes were isolated using mouse CD11b Microbeads (Miltenyi) and MACS LS separation columns (Miltenyi) according to manufacturer’s protocol. After isolation, monocytes were labeled using CFSE (CellTrace™ CFSE Cell Proliferation Kit) according to manufacturer’s protocol.

### Chemotaxis assay or Boyden chamber assay

250,000 monocytes were seeded onto the upper chamber FluoroBlok inserts (Falcon, Fluorescence Blocking PET track-etched membrane 24-well format) in 200 µl, while 700 µl of phenol free RPMI containing either Sema3A or fMLP was filled into the lower one. Monocyte migration was monitored by acquiring six microscopic images per well, avoiding the center and edges of the well. These images were taken at 1, 2 and 4 h for spontaneous migration, and 4 h for comparing WT and HZ monocyte chemotaxis. Images were analyzed using ImageJ software.

### Bone marrow derived macrophages isolation

Both tibia and femurs were collected form Sema3A WT mice in ice cold PBS and stripped from muscles. After placing the stripped bones in 70% Ethanol for approximately 45 s they washed again with PBS after which the ends were cut off and the inner bone marrow was flushed out with a 25G syringe filled with cold PBS. After flushing all the bones a single cell suspension was obtained by pushing the suspension through a 100 µm Nylon cell strainer. Cells were spun down at 1200 rpm and placed in bacterial plates in RPMI 1640 with 15% LCM for culture and differentiation for approximately 8–10 days, adding or replacing medium every 2–3 days. Experiments were performed after differentiation in 8–10 days. Cells were counted using a Burker Turk cell counting chamber and 400,000 cells seeded in 12-wells plates. The cells were stimulated the next day after the cells had adhered to the plastic. At the end of the experiments the cells were directly harvested in RLT buffer for RNA isolation, and sample buffer for immunoblotting.

### Efferocytosis assay

Macrophages (200,000) were seeded in 24-well tissue culture plate and polarized with either 10 ng/ml LPS and 10 ng/ml INF-γ or 10 ng/ml IL-10 with and without presence of 150 ng/ml recombinant Sema3A, produced in a Chinese Hamster Ovary cell line (R&D systems, Cat. No. 5926-S3-025) [25, 34]. Jurkat-cells were cultured in 10 mm dishes in RPMI 1640 with 10% FBS. Jurkat-cells were stained with CFSE (CellTrace™ CSFE (Cat. No. C34554), Life Technologies), according to the protocol supplied by the manufacturer. Briefly, cells were spun down and incubated with CFSE labeling solution (1:1000 in warm PBS) for approximately 15 min at 37 °C. Subsequently, they were re-pelleted and incubated in fresh pre-warmed medium for another 30 min. After washing the cells with PBS they were counted and re-suspended in 10 ml medium in a 10 mm dish. Irradiating the cells for 5 min with 1200 × 100 µJ/cm^2^ induced apoptosis in the Jurkat-cells. To ensure homogenous exposure the plate was swirled every 60 s. The irradiated cells were incubated for another 2 h after which they were seeded in a 1:1 ratio on top of the seeded macrophages and incubated with the polarized macrophages for approximately 45 min. Afterwards they were removed and the macrophages washed twice with ice cold PBS and fixed with 1% paraformaldehyde for 10 min. Cells were then washed again with PBS after which images were taken immediately.

### Statistical analysis

Data were expressed as mean ± SEM. No repeated measurements were performed. Echocardiograph measurements and histological and molecular analysis in sham operated and infarct groups were performed in independent groups. Normal distribution of all continuous variables was tested using the Kolmogorov–Smirnov test. An unpaired *t* test was used in most of the comparisons when groups passed the normality test. However, a Mann–Whitney test was used when the standard deviations of two groups were significantly different. A two-sided *p* value <0.05 was considered statistically significant.

## Results

### Sema3A expression increases during myocardial infarct healing

**Fig. 1:**
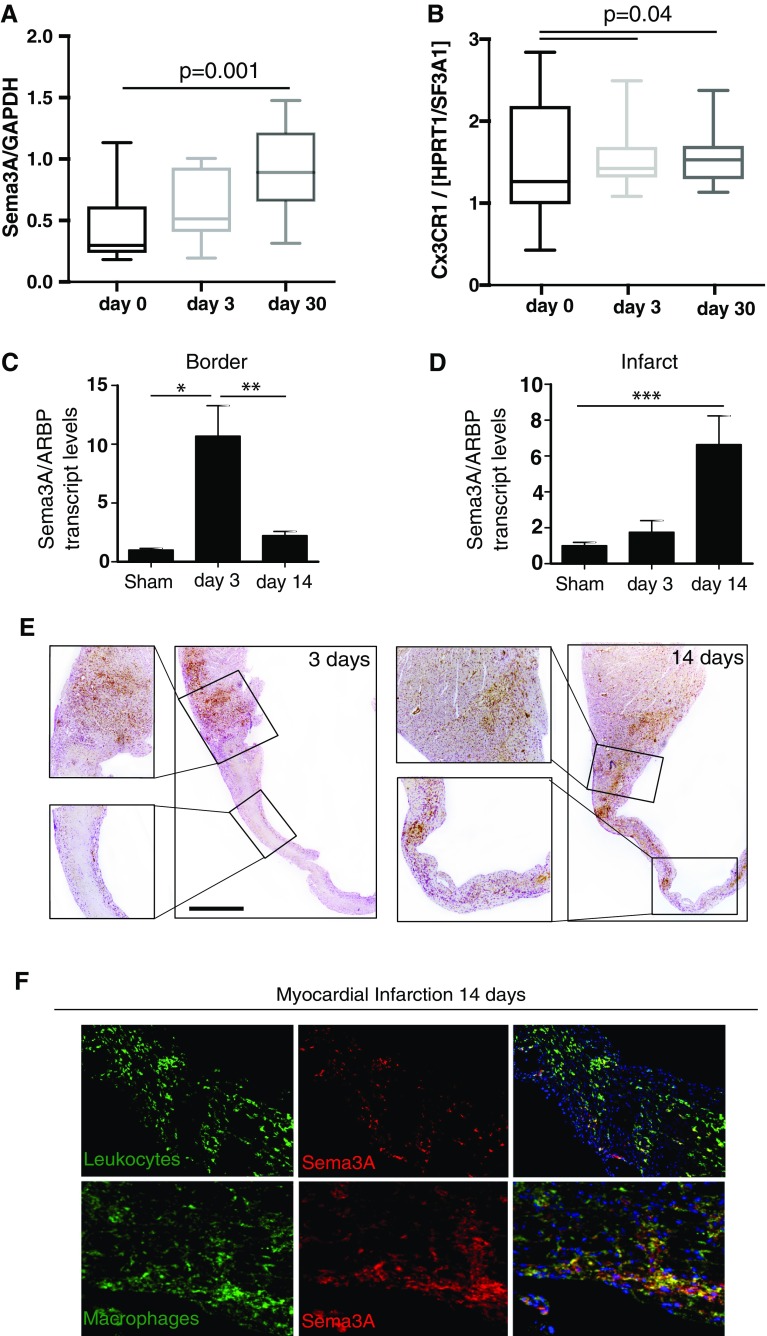
Sema3A expression increases during infarct healing and is present on macrophages. **a**, **b** Increased expression of Sema3A and Cx3CR1 following myocardial infarction in circulating monocytes from patients at day 3 and significant increases at day 30 post admission (*p* = 0.001 and 0.04, *n* = 11). **c** Sema3A gene expression increased significantly in the border zone 3 days after myocardial infarction in the murine model (*p < 0.05, **p < 0.01, *n* ≥ 4). Fourteen days after ischemic injury, the expression also increased in the infarcted area (***p < 0.001, *n* ≥ 4). **d** The expression of Sema3A in both the infarct and border zone was confirmed by immunohistochemistry. Where Sema3A expression predominated in the border zone 3 days after MI, 14 days after MI the increased expression in the infarct became more apparent (**e**). RNA expression decreased to baseline levels in the infarct border zone at 14 days post-MI. Counterintuitively, immuno-histochemical staining still showed substantial Sema3A presence at this time. This apparent discrepancy might be indicative of the slower rate of protein degradation. Immunofluorescence further revealed Sema3A location on leukocytes and macrophages (**f**). *Scale bar* 50 µm

Sema3A gene expression increased progressively in circulating monocytes from patients post myocardial infarction and reached significance at day 30 (Fig. 1a). Interestingly these increases were also observed in the gene expression of Cx3CR1 (Fig. 1b)—a marker of reparative non-classical monocytes in humans [39]. In the murine model of myocardial infarction, 3 days after myocardial infarction Sema3A gene expression increased significantly in the areas bordering the infarcted tissue (*p* < 0.05), returning back to baseline levels after 14 days (Fig. 1c, *p* < 0.005). In the infarcted area expression of Sema3A transcripts increased significantly at 14 days after infarction (Fig. 1d, *p* < 0.001). Concomitantly, Sema3A expression was observed predominantly in the border zone and not in the infarcted area at 3rd day (Fig. 1e), whilst at 14th day Sema3A expression was concentrated within the infarct (Fig. 1f). Finally, Sema3A was detected on infiltrated leukocytes (CD45^+^ cells) and specifically on the resident macrophage population (MAC-3+ve cells).

### Sema3A reduces cardiac inflammation after myocardial injury

**Fig. 2:**
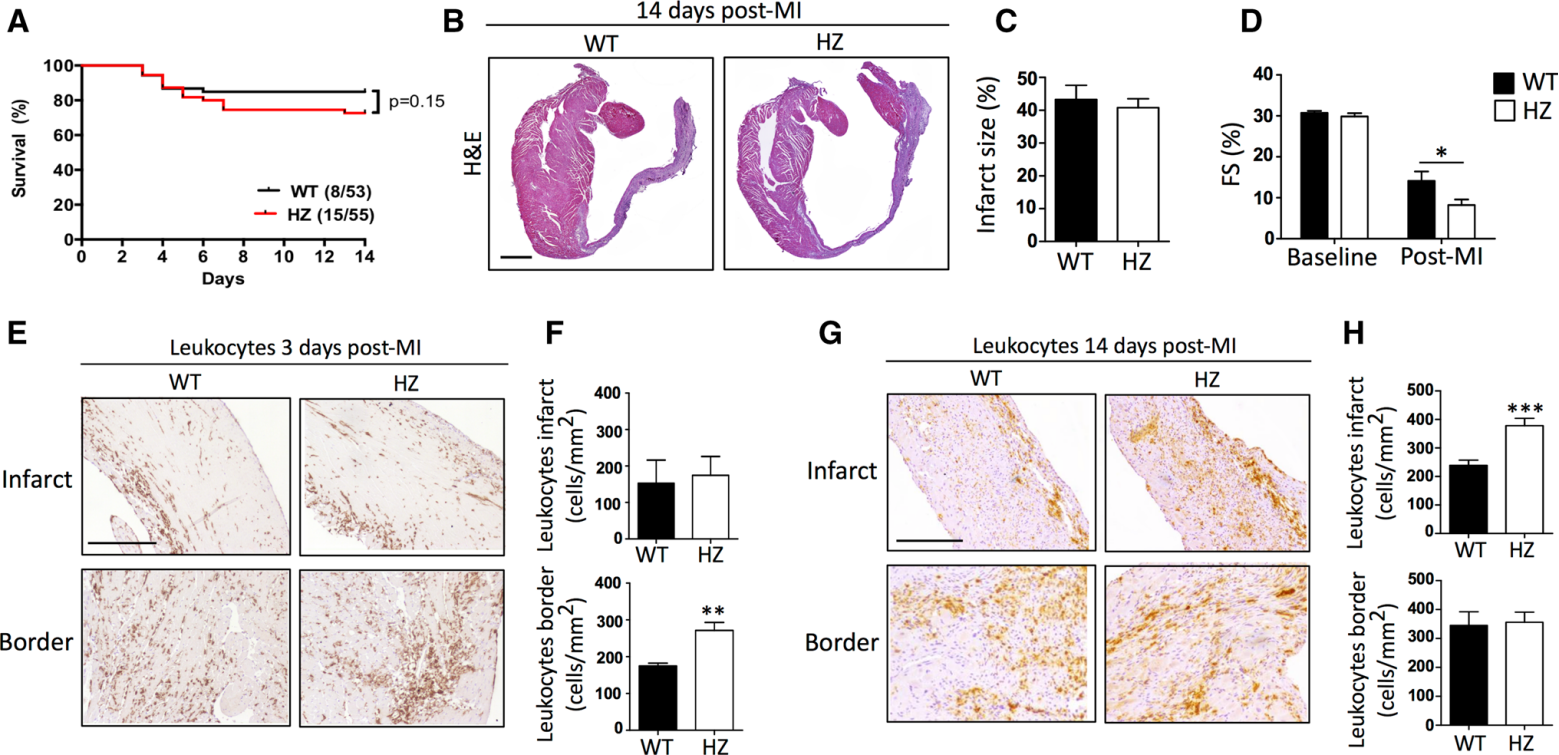
Sema3A regulates inflammation during infarct healing. **a** Coronary artery occlusion in Sema3A WT and HZ mice did not result in a difference in mortality. **b**, **c** Despite comparable infarct sizes between both groups, Sema3A HZ mice showed worse cardiac function with decreased fractional shortening (**d**, *p < 0.05, *n* ≥ 13). The number of recruited immune cells was determined by immuno-histochemical staining for both the infarct and the border zone, 3 and 14 days after MI. **e** Sema3A HZ had more leukocytes in the border zone 3 days after ischemic injury as compared to WT (**p < 0.01, *n* ≥ 4). **f** Fourteen days after infarction the increased presence of leukocytes in Sema3A HZ mice was also apparent in the infarct area (***p < 0.001, *n* ≥ 9). All experiments were repeated at least twice

Sema3A knockout mice are embryonically lethal yet the heterozygous mice (HZ) are viable and express approximately half the amount of Sema3A mRNA at baseline in the heart in comparison to their wild type (WT) littermates (1.22 ± 0.05 in WT compared to 0.62 ± 0.11 in HZ, arbitrary units) [3]. Sema3A WT and HZ mice were subjected to the mouse model of myocardial infarction (Fig. 2a). Though mortality and infarct size between both genotypes were similar (Fig. 2a–c), fractional shortening was significantly reduced in Sema3A HZ as compared to WT mice (Fig. 2d; Table 1; *p* < 0.05). Furthermore, no significant differences in collagen content, cardiomyocyte hypertrophy or capillary density were seen within the infarcted area between genotypes (Table 2). However, the amount of CD45-positive leukocytes was significantly greater in Sema3A HZ than in WT mice in the border zone at 3rd day, but did not differ in the infarct zone (*p* < 0.005, Fig. 2e, f). Inversely, at 14 days post-MI a significant greater number of CD45-positive leukocytes were observed in the infarct zone of Sema3A HZ mice as compared to WT mice, but not in the border (*p* < 0.001, Fig. 2g, h). A similar increase in inflammation was seen in Sema3A HZ mice compared to WT when using the non-sterile model for cardiac injury of Coxsackie B3 induced viral myocarditis, where Sema3A expression could also be found on macrophages (Supplementary Figure 1). Finally, more macrophages were present in the infarcted area of the Sema3A HZ mice as compared to WT mice at 14th day (*p* < 0.005; Table 2).

**Table 1:** Echocardiography at baseline and 14 days post-MI

	Sham		14 days post-MI	
	WT (*n* = 8)	HZ (*n* = 10)	WT (*n* = 13)	HZ (*n* = 14)
IVSd (mm)	0.8 ± 0.02	0.8 ± 0.04	0.6 ± 0.06	0.5 ± 0.05^£^
LVPWd (mm)	0.8 ± 0.02	0.8 ± 0.02	0.8 ± 0.04	0.7 ± 0.04
LVIDd (mm)	3.9 ± 0.07	3.9 ± 0.07	4.9 ± 0.2^£^	4.6 ± 0.1^£^
LVIDs (mm)	2.7 ± 0.05	2.8 ± 0.07	4.1 ± 0.2^£^	4.0 ± 0.1^£^
FS (%)	30 ± 0.5	30 ± 0.8	17 ± 1.9^£^	11 ± 1.6*^,£^
Heart rate (bpm)	543 ± 55	576 ± 27	580 ± 22	597 ± 15

*IVSd* interventricular septum diastole, *LVPWd* left ventricular posterior wall diastole, *LVIDd* left ventricular internal diameter diastole, *LVIDs* left ventricular internal diameter systole, *FS* fractional shortening, *MI* myocardial infarction

^£^ *p* < 0.05 sham vs. MI

* *p* < 0.05 Sema3A WT vs. HZ post-MI

**Table 2:** Biometric and histological data at baseline, 3 and 14 days post-MI in the infarct zone

	Sham		3 days post-MI		14 days post-MI	
	WT (*n* = 7)	HZ (*n* = 8)	WT (*n* = 9)	HZ (*n* = 9)	WT (*n* = 9)	HZ (*n* = 10)
HW/BW (mg/kg)	4.6 ± 0.2	4.5 ± 0.2	5.2 ± 0.3	6.0 ± 0.4	5.1 ± 0.3	5.3 ± 0.2
Lung/BW (mg/kg)	7.0 ± 0.5	7.6 ± 1.0	7.8 ± 0.3	8.5 ± 0.5	10.0 ± 1.2	9.1 ± 0.7
Infarct size (%)	n.a.	n.a.	45 ± 2.3	42 ± 3.1	52 ± 3.4	51 ± 3.2
Infarct thickness (µm)	n.a.	n.a.	569 ± 32	600 ± 74	229 ± 16	171 ± 12*
Collagen (%)	n.a.	n.a.	n.a.	n.a.	60 ± 2	61 ± 3
Microvessels (vessels/mm^2^)	n.a.	n.a.	n.a.	n.a.	239 ± 34	231 ± 7
Leukocytes (cells/mm^2^)	48 ± 11	42 ± 12	153 ± 64	175 ± 52	239 ± 18	378 ± 26***
Macrophages (cells/mm^2^)	n.a.	n.a.	n.a.	n.a.	230 ± 62	481 ± 42*
Myocyte surface area (µm^2^)	n.a.	n.a.	n.a.	n.a.	339 ± 20	338 ± 18

*HW/BW* heart weight/body weight, *Lu/BW* lung weight/body weight, *MI* myocardial infarction, *n.a.* not applicable

* *p* < 0.05 Sema3A WT vs. HZ post-MI

*** *p* < 0.0001 Sema3A WT vs. HZ post-MI

### Sema3A does not induce a difference in monocyte activation and recruitment

**Fig. 3:**
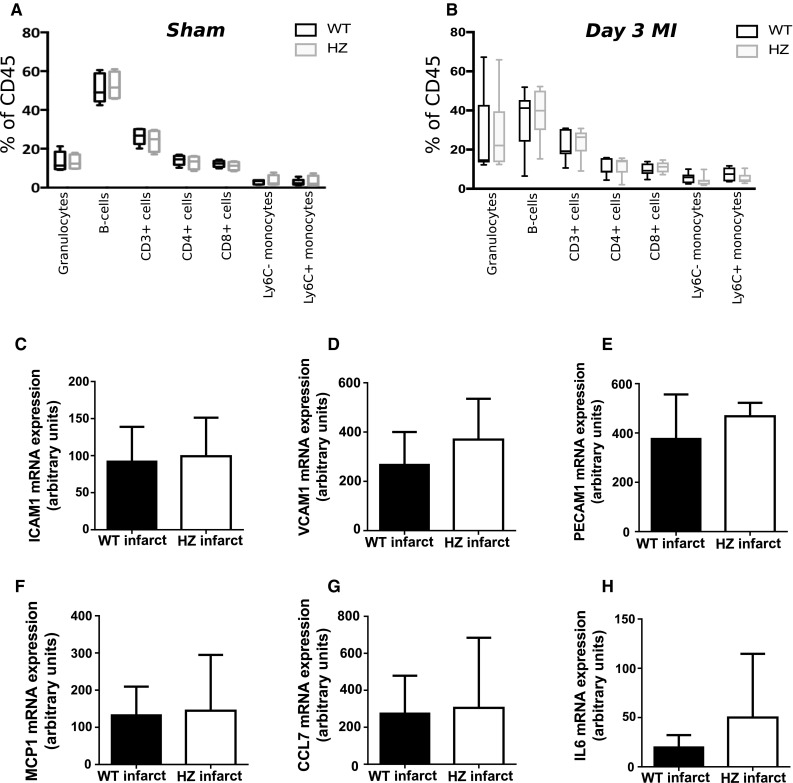
Sema3A does not regulate leukocyte activation, adhesion molecule and chemokine expression central to leukocyte transmigration. **a** Flow cytometric analysis of circulation leukocytes did not reveal any differences in the distribution of leukocyte subsets in the Sema3A HZ mice (*n* = 4) as compared to WT mice (*n* = 5). **b** There were no differences between Sema3A HZ and WT mice in the number of leukocytes in the circulation 3 days after myocardial infarction (*n* = 4 vs. 6, respectively). **c**–**e** RNA expression of adhesion molecules VCAM1, ICAM1 and PECAM1 is similar in Sema3A WT compared to HZ mice 14 days after myocardial infarction (*p* = ns, *n* ≥ 6). **f**–**h** Further, equal RNA expression of monocyte chemoattractant MCP1 and CCL7 and pro-inflammatory cytokine IL6 was found in Sema3A WT compared to HZ mice 14 days after myocardial infarction (*p* = ns, *n* ≥ 6)

Flow cytometric analysis was performed to assess the distribution of leukocytes in the circulation in the Sema3A HZ mice as compared to WT mice without cardiac injury, with no significant changes observed (Fig. 3a). Furthermore, decreased Sema3A levels did not result in significant changes in the amount of leukocyte subsets present in the circulation 3 days after myocardial infarction (Fig. 3b). As increased monocytosis could not explain the increased presence of macrophages in the infarcted area, the cardiac expression of adhesion molecules and chemokines known to influence monocyte recruitments were measured in Sema3A HZ and WT mice. No differences were found in the expression of adhesion molecules VCAM1, ICAM1 and PECAM1 between Sema3A WT and HZ mice (Fig. 3c–e). Furthermore, monocyte chemoattractant MCP1 and CCL7 as well as pro-inflammatory cytokine interleukin-6 did not show any differences in mRNA expression between both genotypes (Fig. 3f, g).

### Sema3A crucial for constraining monocyte recruitment and inducing pro-inflammatory macrophage apoptosis

**Fig. 4:**
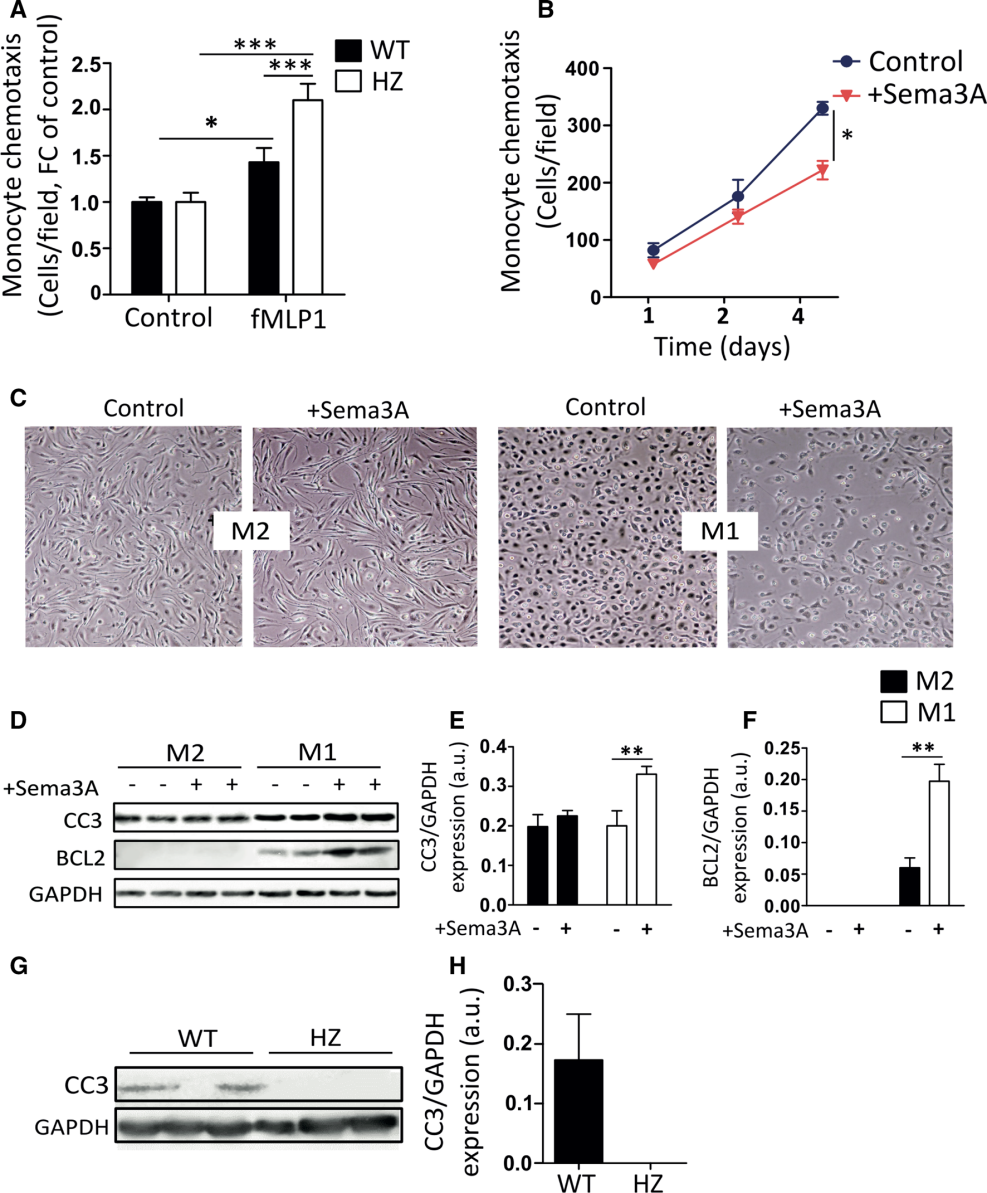
Sema3A inhibits chemotaxis and promotes pro-inflammatory macrophages clearance by inducing apoptosis. The migratory capacity of isolated splenic monocytes was assessed using a Boyden Chamber Assay for chemotaxis. **a** The migratory capacity of Sema3A WT and HZ monocytes towards the chemoattractant fMLP1 revealed the decreased migratory capacity in Sema3A WT monocytes as compared to HZ (*p < 0.05, ***p < 0.001, *n* = 4). **b** Sole presence of Sema3A in the lower chamber significantly confirmed decreased migration by inhibiting spontaneous migration of monocytes (*p < 0.05, *n* = 4). **c** Bone marrow derived macrophages were isolated and polarized for 24 h in the presence of either IL-10 or LPS in combination with INFγ towards M2 and M1, respectively. Stimulation of M1 with Sema3A resulted in a decreased cell density as compared to M2 cells. Western blot analyses (**d**) and quantification (**e**, **f**) for Cleaved Caspase 3 (CC3) and BCL-2 revealed the decreased cell density in the M1 as a consequence of increased apoptosis (**p < 0.01, *n* = 3). Western blot analysis (**g**) and quantification (**h**) revealed clear presence of CC3 in WT myocardial infarcts that could not be detected in HZ mice (*n* = 6). All experiments were repeated at least twice

In vitro chemotaxis experiments explored whether Sema3A itself could regulate monocyte migration. Isolated primary monocytes from HZ mice migrated significantly faster towards the chemoattractant fMLP than WT monocytes (Fig. 4a; *p* < 0.001). In parallel, the presence of recombinant Sema3A protein in the bottom of a Boyden chamber impeded the migration of WT monocytes (*p* < 0.05; Fig. 4b). We further addressed the effect of Sema3A on macrophage function, as timely resolution of cardiac inflammation is a vital step in proper cardiac healing in response to ischemia. Therefore, bone marrow derived macrophages were polarized towards an anti-inflammatory (alternative-Mϕ, M2) phenotype or towards a pro-inflammatory (classical-Mϕ, M1) phenotype [27], prior to stimulation with recombinant Sema3A. Stimulation with recombinant Sema3A of polarized macrophages resulted in a visible decrease in cell density in the pro-inflammatory classical-Mϕ (M1), but not in the alternative-Mϕ (M2) (Fig. 4c). Furthermore, stimulation with Sema3A significantly increased the levels of apoptotic markers cleaved caspase-3 (CC3) and BCL2 in the classical-Mϕ, but not in alternative-Mϕ (Fig. 4d, e; *p* < 0.01). Concomitantly, Sema3A HZ mice had no detectable CC3 protein expression in the infarcted area, which was detectable in the infarcts of WT mice at 14th day post-MI (Fig. 4f).

### Sema3A promotes the transition of classically activated macrophages towards resolution

**Fig. 5:**
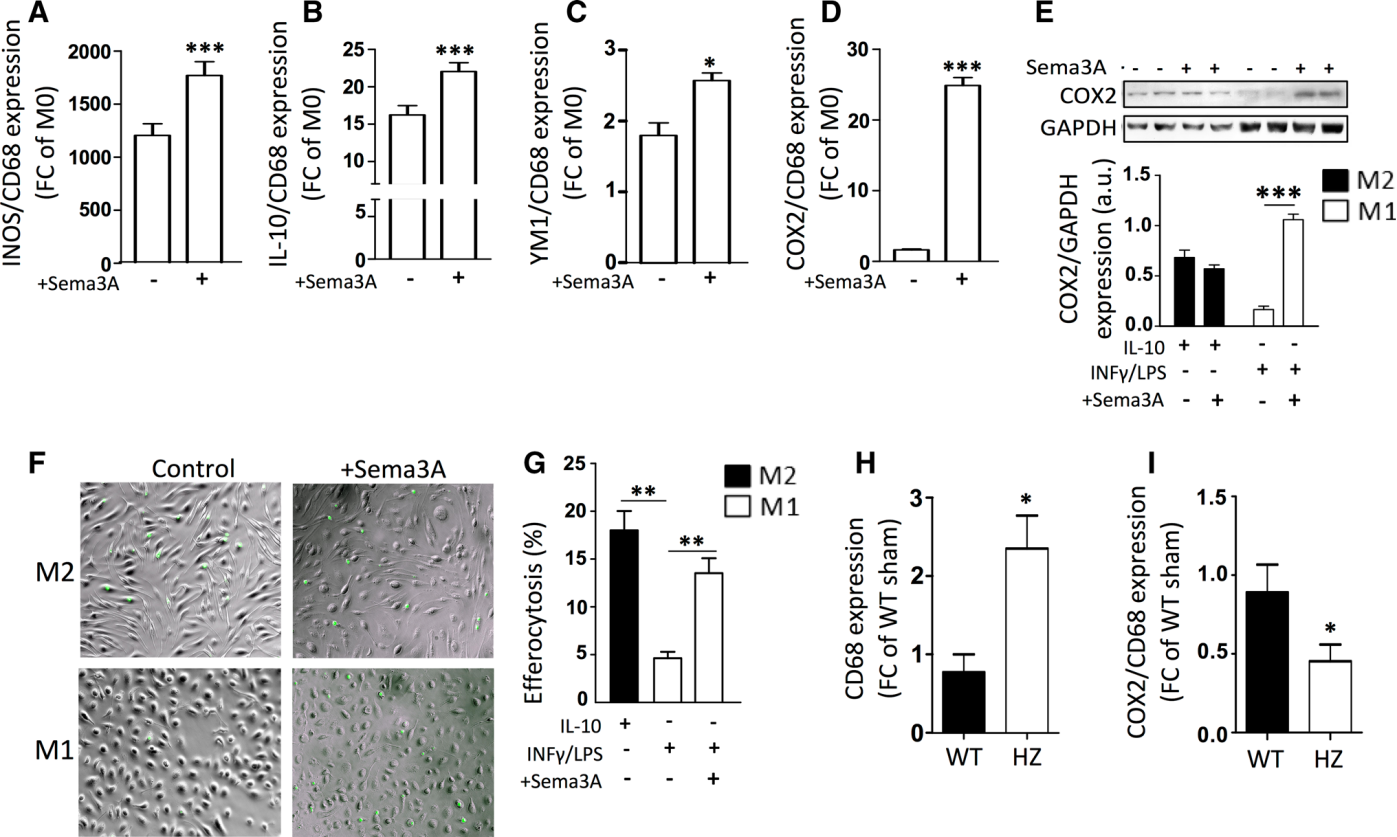
Immune resolution is promoted by Sema3A via selective induction of a phenotypic switch in pro-inflammatory macrophages towards resolution-phase-macrophages. Bone marrow derived macrophages were isolated and polarized for 24 h towards M1 with and without the presence of Sema3A. **a**–**c** The expression of iNOS, IL-10 and YM1 increased significantly in response to stimulation with Sema3A (*p < 0.05, ***p < 0.001, *n* = 3). Interestingly, the addition of Sema3A resulted in a marked induction of COX2 transcript levels (**d**) and protein levels (**e**) (***p < 0.001, *n* = 3). Clearance of apoptotic cells by means of efferocytosis was assessed in vitro. Fluorescently labeled apoptotic Jurkat-cells were fed to M2 and M1 with and without the presence of Sema3A. As expected, M2 were significantly better at clearing apoptotic Jurkat-cells than M1 (**f**, **g**). Remarkably, addition of Sema3A to M1 resulted in a significant increase in the percentage of efferocytotic cells (**f**, **g**) (**p < 0.01, *n* = 3). All experiments were repeated at least twice

Resolution-phase macrophages are neither classical-Mϕ nor alternative-Mϕ, but a hybrid of both, and are characterized by the expression of inducible cyclooxygenase (COX2) and iNOS [4]. Stimulation of classical-Mϕ (M1) with recombinant Sema3A significantly increased the transcript expression of Mϕ-markers iNOS, IL-10, YM-1 and COX-2 (Fig. 5a–d). Furthermore, Sema3A stimulation increased COX2 protein levels in the pro-inflammatory classical-Mϕ (M1), but not in the alternative-Mϕ (M2, Fig. 5e). To demonstrate that Sema3A not only shifts the phenotype of classical-Mϕ towards ‘resolution-Mϕ’, the function of these macrophages to clear inflammation (efferocytosis)—a crucial step in correct infarct healing—[38] was assessed. Alternative-Mϕ are better at clearing dying cells than classical-Mϕ; however, in the presence of recombinant Sema3A classical-Mϕ increased significantly their efferocytotic capabilities (Fig. 5f, g; *p* < 0.01). In parallel, COX2 levels were significantly lower in infarcted cardiac tissue of Sema3A HZ as compared to WT mice, despite higher levels of CD68 (Fig. 5h, i; *p* < 0.05).

## Discussion

The present study demonstrates that Sema3A is important for proper myocardial wound healing. It is the first study to demonstrate a role for Sema3A in promoting the resolution of inflammation after AMI which may be in line with the increased expression of Sema3A in monocytes of human subjects following myocardial infarction. The recruitment of monocytes is of critical importance in the wound healing response following myocardial injury, but restraining the flow of monocytes is equally needed to avoid excessive inflammation that may contribute to adverse cardiac remodeling [13, 20]. Sema3A regulates the inflammatory phase following myocardial injury by curbing the flow of recruited cells, but also by promoting the transition of classical-Mϕ to resolution or alternative-Mϕ.

**Fig. 6:**
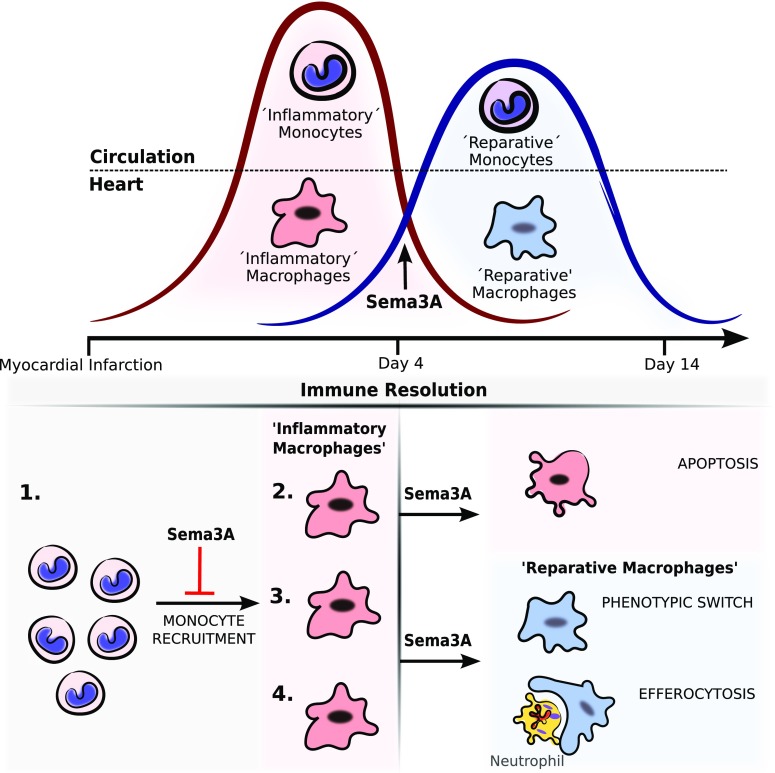
Sema3A promotes immune resolution. Schematic overview of how Sema3A promotes immune resolution on four different levels. Presence of Sema3A in the border zone (*1*) prohibits excessive immune cell recruitment to the infarct. (*2*) Sema3A in the infarct promotes the clearance of inflammation by inducing apoptosis in M1 macrophages (*3*), promotes a phenotypic shift of M1cells towards resolution (Mr) and (*4*) enhances efferocytosis in M1 cells

Recently, Hou et al. demonstrated that Sema3A induces vascular permeability in cerebral ischemia-induced brain damage via the neuropilin-2/VEGR1 receptor complex [21]. In addition, Sema3A also competes with VEGF, hence affects vascular permeability during angiogenesis [1]. Despite cardiac ischemia initiating changes in vascular permeability and angiogenesis, the reduced Sema3A in the HZ mice did not affect these processes. Sema3A has not previously been ascribed a role in resolving inflammation, though Sema3A has been shown to limit the recruitment of tumor-associated macrophages, restricting angiogenesis and restoring antitumor immunity [5]. In line with previous work on Sema3A and macrophages [22], this study reveals that Sema3A promotes the resolution of cardiac inflammation by regulating monocyte/macrophage function in four ways: (1) limiting the recruitment of monocytes, (2) promoting the apoptosis of classical-Mϕ (M1) and (3) stimulating their transition to resolution-phase macrophages, thereby (4) enhancing efferocytosis (Fig. 6). Though it is accepted that alternative-Mϕ, along with resolution-Mϕ are critical for the transition to repair, the authenticity of well-defined M1 and M2 macrophage populations in vivo is still of ongoing debate [28]. Still, while little is known about the local factors that mediate the shift to repair, recently IRF5 and NR4a-1 were shown to be critical in promoting the transition of macrophages towards resolution [11, 18]. However, more is known about the signals coming from T-cell subsets that promote repair such as IL-13 [19] and thymosin beta4 [14]. Here we show that Sema3A is vital for regulating cardiac monocyte/macrophage function in response to ischemic induced myocardial injury. Despite additional experiments, we were not able to provide substantial evidence that will allow us to ascribe the effect of Sema3A in response to ischemic induced myocardial injury exclusively to monocytes/macrophages. By encouraging a shift towards immune resolution, Sema3A helps to maintain cardiac function. Sema3A may, therefore, be of therapeutic value not only as an anti-inflammatory agent in autoimmune diseases and cancer, but potentially in preventing the development of heart failure by limiting adverse cardiac remodeling.

## Electronic supplementary material

Below is the link to the electronic supplementary material.

Supplementary material 1 (PDF 64 kb)
