# Supplementary figures and images for: Sema3A promotes the resolution of cardiac inflammation after myocardial infarction
Source: Basic Res Cardiol. 2017 May 24;112(4):42. doi: 10.1007/s00395-017-0630-5 (PMC5443852; doi:10.1007/s00395-017-0630-5)

Supplementary Figure 1

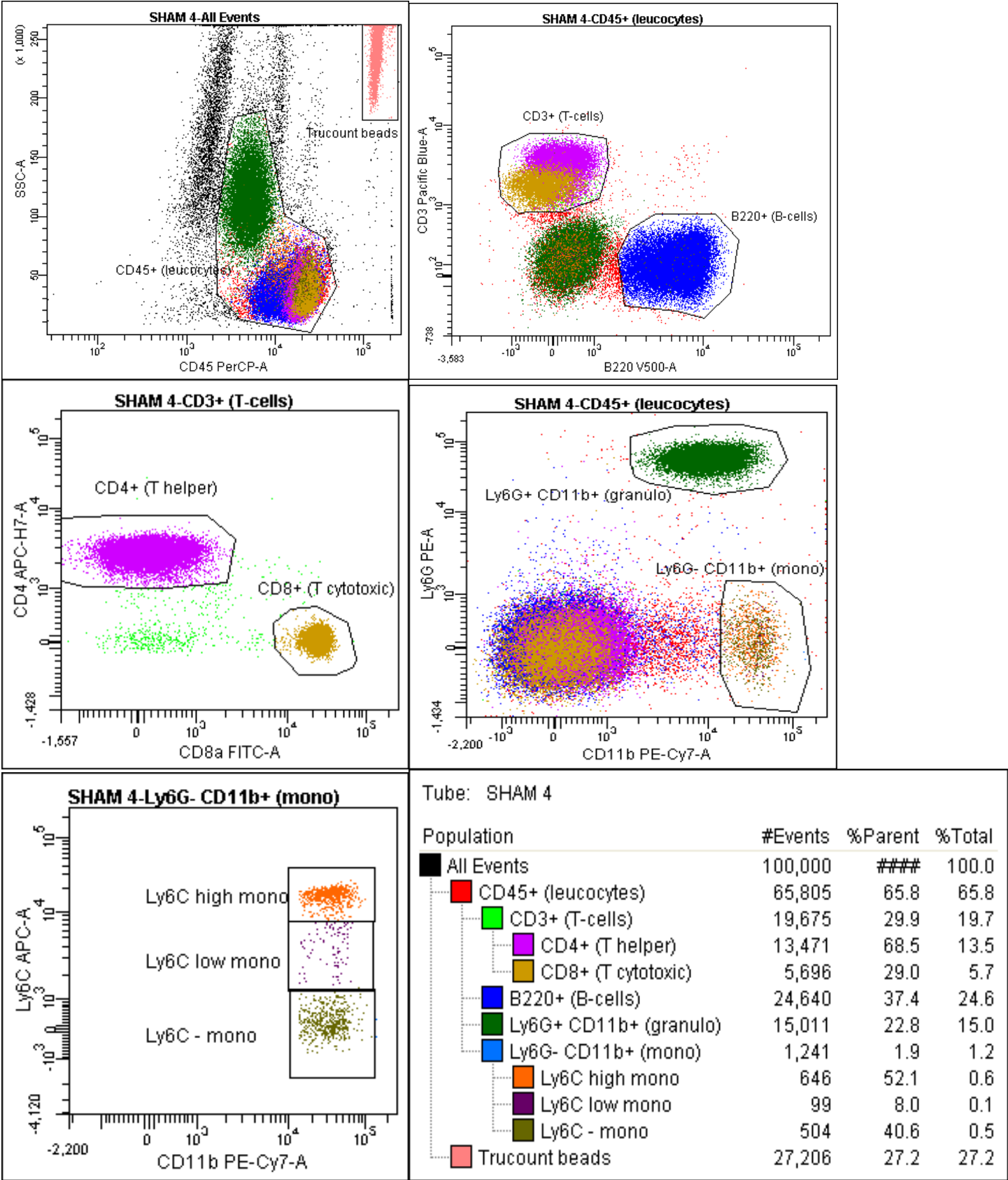

Supplement: Supplementary file 1 — Supplementary material 1 (PDF 64 kb) [file 395_2017_630_MOESM1_ESM.pdf]
